# Detection and identification of Bogia coconut syndrome phytoplasma from seed-associated tissues and seedlings of coconut (*Cocos nucifera*) and betel nut (*Areca catechu*)

**DOI:** 10.1038/s41598-024-61916-4

**Published:** 2024-05-21

**Authors:** Hengyu Lu, Bree Wilson, Hanfang Zhang, Sharon B. Woruba, Bowen Feng, Anne C. Johnson, Birte Komolong, Lastus Kuniata, Guang Yang, Geoff M. Gurr

**Affiliations:** 1 State Key Laboratory of Ecological Pest Control for Fujian and Taiwan Crops, Institute of Applied Ecology, Fujian Agriculture and Forestry University, Fuzhou, 350002 China; 2 Key Laboratory of Integrated Pest Management for Fujian-Taiwan Crops, Ministry of Agriculture, Fuzhou, 350002 China; 3 Key Laboratory of Green Control of Insect Pests (Fujian Agriculture and Forestry University), Fujian Province University, Fuzhou, 350002 China; 4 Fujian-Taiwan Joint Centre for Ecological Control of Crop Pests, Fujian Agriculture and Forestry University, Fuzhou, 350002 China; 5 Centre for Crop Health, Institute for Life Sciences and the Environment, University of Southern Queensland, Toowoomba, QLD Australia; 6 Kokonas Indastri Koporesen, Madang, Madang Province Papua New Guinea; 7 Gulbali Institute, Charles Sturt University, Orange, NSW 2800 Australia; 8 National Agricultural Research Institute, P.O. Box 4415, Lae, Morobe Province Papua New Guinea; 9 New Britain Palm Oil, Ramu Agri Industries Ltd, Gusap Downs, PO Box 2183, Lae, Morobe Province Papua New Guinea

**Keywords:** Seed borne, Seed transmission, Lethal yellowing disease, 16S rRNA, Ecology, Microbiology, Plant sciences

## Abstract

Evidence for seed transmission of phytoplasmas has grown in several pathosystems including coconut (*Cocos nucifera*). Bogia coconut syndrome (BCS) is a disease associated with the lethal yellowing syndrome associated with the presence of ‘*Candidatus* Phytoplasma noviguineense’ that affects coconut, betel nut (*Areca catechu*) and bananas (*Musa* spp.) in Papua New Guinea. Coconut and betel nut drupes were sampled from BCS-infected areas in Papua New Guinea, dissected, the extracted nucleic acid was used in polymerase chain reaction (PCR), and loop mediated isothermal amplification (LAMP) used to check for presence of phytoplasma DNA. In a second study, drupes of both plant species were collected from multiple field sites and grown in insect-proof cages. Leaf samples taken at 6 months were also tested with PCR and LAMP. The studies of dissected coconut drupes detected phytoplasma DNA in several tissues including the embryo. Drupes from betel nut tested negative. Among the seedlings, evidence of possible seed transmission was found in both plant species. The results demonstrate the presence of ‘*Ca*. P. noviguineense’ in coconut drupes and seedlings, and in seedlings of betel nut; factors that need to be considered in ongoing management and containment efforts.

## Introduction

Seed transmission of phytoplasmas has been identified in several plant species through the detection of phytoplasma DNA in plant embryos and seedlings^1,2^. Plant to plant transmission of these phloem-limited pathogens is primarily done by phloem-feeding hemipteran insects^3,4^, vegetative propagation or grafting and, in some crops, via the parasitic plant *Cuscuta* spp.^5,6^. Bogia Coconut Syndrome (BCS) (‘*Candidatus* Phytoplasma noviguineense’) in Papua New Guinea (PNG) is associated with Coconut Lethal Yellowing disease^7^. Elsewhere in the world, where other coconut lethal yellowing diseases have occurred, the impacts have been so severe that there is urgency to understand how BCS might be spread. This will ensure that containment measures are evidence-based and thereby economic and social upheaval is minimised in PNG^8^.

Phytoplasma colonization takes place in the plant phloem and seed transmission was initially considered unlikely because there is no direct connection to developing seeds from phloem sieve elements^1,9^. However, phytoplasmas have been shown to be able to reach other plant tissues especially floral structures^10^, including the stems, racemes, male and female flowers and embryos of palms^11,12^. Doubts about seed transmission of phytoplasmas were also due to the host plant’s compromised ability to viably reproduce, due to malformation, withering and reduced seed size^1^, and in coconut (*Cocos nucifera*), premature dropping of immature coconuts^7^. The viability of seed is dependent on the timing of host plant infection and the length of time it takes for disease symptoms to affect plant reproduction. In coconut, the time from pollination to maturity of the fruit is around 12 months, and in the Caribbean, this was also found to be the time for developing disease symptoms of Coconut Lethal Yellowing phytoplasma; therefore, the fruit at the time of infection could be still ripening^9^. Recent studies have demonstrated the presence of phytoplasmas from other 16Sr groups in the seed from infected plants or seedlings grown from such seeds including alfalfa (*Medicago sativa* L.)^13^, pea (*Pisum sativum* L.)^14^, oilseed rape (*Brassica napus* L.), tomato (*Solanum lycopersicum* L.), corn (*Zea mays* L.)^2,15^, and carrot (*Daucus carota* L.)^16^.

Phytoplasma-positive results from coconut embryos using nested PCR and in situ PCR^9^ led to international legislation to prohibit commercial movement of coconuts from areas where coconut lethal yellowing is epidemic^1^. The occurrence of 16SrXI group phytoplasmas associated with root wilt disease in coconut embryos has been reported in India^17^. In Ghana, 16SrXXII phytoplasma associated with Cape St Paul wilt disease was detected in multiple plant parts including embryos, but no evidence presence of the phytoplasma was found in germinated seedlings^11^. Detection of the 16SrIV lethal yellowing phytoplasma has been reported for Mexican coconut plantlets through in vitro germination of zygotic embryos^18^.

The preceding evidence is consistent with the possibility of transmission of coconut phytoplasmas by seed but there is no evidence demonstrating that seeds collected from the field contain viable phytoplasma pathogens and generate infected plants under field conditions. More specifically, this study is the first to examine the possibility of seed transmission for ‘*Ca.* P. noviguineense’. This study was carried out in hotspots of BCS, collecting drupes directly from coconut and betel nut (*Areca catechu* L.) palms. Betel nut is common in BCS-affected areas and was included in the study because it represents a potential means of spread since the unprocessed drupes of this plant are widely traded in PNG. Another phytoplasma, Banana Wilt associated phytoplasma (BWAP) is also found in the BCS region and is genetically undistinguishable from to the BCS-associated phytoplasma^19,20^. With distinctive yellowing and necrosis, BWAP is found around Madang and also in a number of regions across PNG.

In this research, tissue samples from coconut husk, shell, flesh, embryo and juice were tested for phytoplasma DNA by using nested PCR. A second study collected drupes from symptomatic and non-symptomatic coconut and betel nut palms. Drupes were placed in insect proof cages until seeds germinated. Leaf and petiole sampling of the resulting seedlings was performed six months after germination. DNA was extracted from the tissue samples and tested for DNA presence using nested PCR, Loop mediated isothermal amplification (LAMP) and sequencing.

## Materials and methods

### Sampling for plant tissue testing

**Figure 1:**
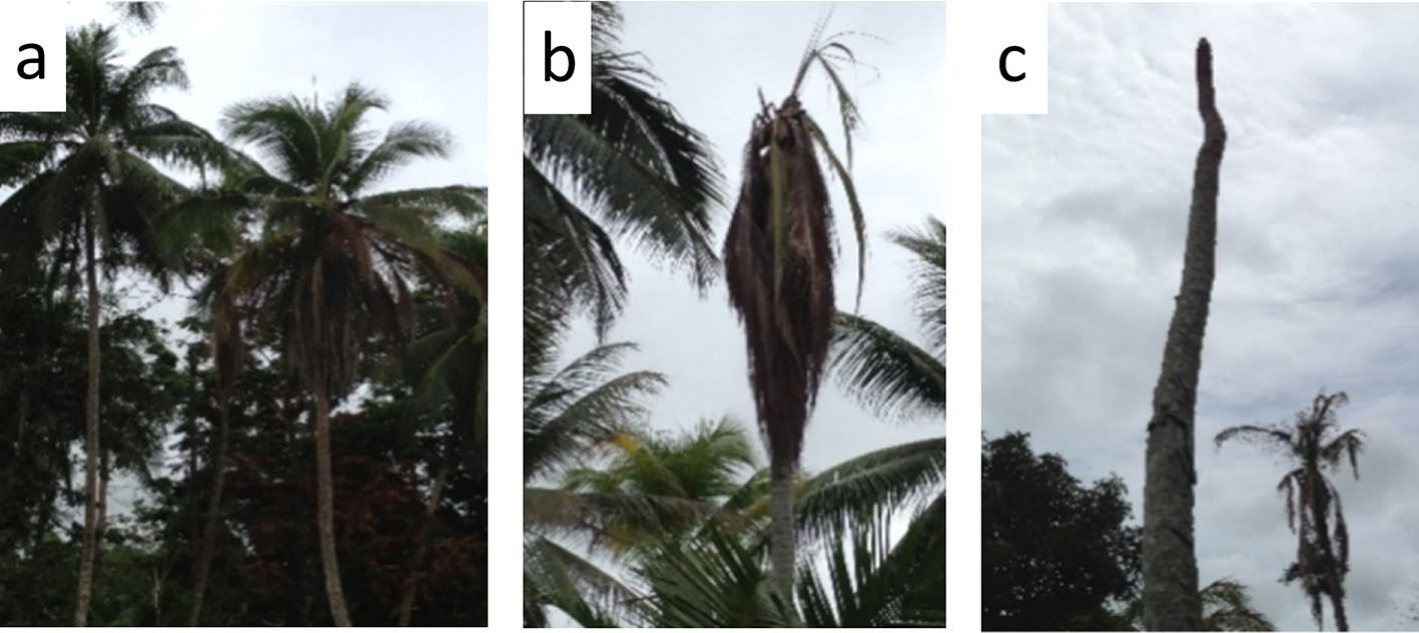
Stages in symptoms of Bogia coconut syndrome in coconut palms (after Miyazaki et al. 2018). (**a**): stage 1, ascending chlorosis and necrosis of fronds progressing to fall of immature drupes); (**b**): stage 2, crown reduced to youngest ‘spear’ leaf and remaining fronds hanging (mature nuts can be present); (**c**): stage 3, bare trunk of dead palm. (Photos: Geoff Gurr).

Field sampling of coconuts involved the collection of 30 drupes (coconuts) and 30 drupes from betel nut (nuts) from palms located in Madang Province, PNG^21^. Ten drupes of each plant species were collected from each of three BCS-affected sites at Mobdu, Siar and Bogia. Collection of plant material complied with relevant guidelines and was conducted in coordination with the National Agriculture Quarantine and Inspection Authority of PNG. Plants were identified by S.B. Woruba of the Kokonas Indastri Korporesen (KIK), formerly the Cocoa and Coconut Institute. Voucher specimens of plant material were not made but DNA samples from each palm are available on request from the corresponding author. At each site, five palms from each species were selected and two fruits were collected from each palm. At each site, two of the coconut palms exhibited severe stage-1 BCS symptoms while the remaining three palms had stage-2 symptoms (Fig. 1). None of the betel nut palms exhibited obvious symptoms. Coconut drupes were dissected to obtain samples of husk (exocarp), shell (endocarp), flesh (endosperm), embryo and milk (liquid endosperm). Structurally simpler betel nut drupes were dissected to obtain tissue samples of exocarp and endosperm. After dissecting separate tissues for a given drupe, the knife was flame sterilized to avoid cross contamination. Tissue samples were placed individually into 100% ethanol in labelled 10 ml vials and transported to the laboratory then stored at 4 °C in a fridge with uninterrupted power awaiting further testing.

### Collections for seed transmission testing

An additional field collection of coconut drupes (n = 39) and betel nut drupes (n = 60) was made. Coconuts were collected from symptomatic palms at four BCS hotspots (Muad village, Oad village/Bilbil, Rivo village area, and Sugar village north of Matukar) in the Madang Province. Coconuts were also collected from non-symptomatic palms (cv. Karkar Tall) located in the Genebank collection at the Murunas Research Station, at KIK, Madang. Betel nut drupes were from two BCS symptomatic palms located at Mobdu village, where coconut palms commonly exhibited BCS symptoms. Coconut and betel nut drupes were grown in individual pots located within insect-proof cages. Germinated seedlings were repeatedly treated with a mixture of fungicide and insecticide. Leaves and petioles were sampled six months after germination and the extracted DNA was analyzed for the presence of ‘*Ca*. P. noviguineense’ using nested PCR and LAMP.

### DNA extraction and nested PCR detection

Genomic DNA was extracted from each sample using the Qiagen DNeasy Mini Plant Kit. A nested PCR was run using primers R16mF2/R1 followed by R16F2n/R2 which are universal for phytoplasmas^21,22^ using a Bio-Rad T100 thermal cycler. PCR products initially amplified were diluted to 1/20 with sterile distilled water as the template for the nested PCR amplification. The reaction mixture was adjusted to 25 µl reactions using sterile distilled water, consisting of 1 µl of 10 µM each primer, 12.5 µl of 2 × GoTaq Green Master Mix (Promega) and 1 µl of DNA template . After an initial denaturation at 94 °C for 2 min, amplification was carried out for 35 cycles with the following parameters: denaturation at 94 °C for 30 s, annealing at 60 °C for 30 s (55 °C for the nested PCR), and extension at 72 °C for 3 min, followed by a final extension at 72 °C for 10 min. DNA extracted from BCS symptomatic *C. nucifera* leaf tissue (RID5162) served as a positive control for all reactions. Chi-square tests were used to compare the incidence of phytoplasma-positive detections among different tissues using IBM SPSS statistics 20.

### Loop mediated isothermal amplification (LAMP)

Two LAMP assays were used to test for the presence of phytoplasmas^22^. Briefly, LAMP primers were designed on the partial sequence of the 16S ribosomal RNA gene; 16S-23S ribosomal intergenic spacer region and partial sequence of the 23S ribosomal gene of *C. nucifera* symptomatic for a BCS strain (GenBank accession number KP053907) using LAMP Designer 1.13 (Premier Biosoft International). The Phyto1 LAMP primer set was designed to detect a range of phytoplasmas including BCS and groups such as 16SrII, 16SrXIV) and a second more specific that aimed to detect only BCS and BWAP by amplification of the intergenic spacer region. DNA from phytoplasma RID5162 was amplified using universal primers P1/P7^23,24^. After an initial denaturation at 94 °C for 90 s, amplification was carried out for 30 cycles with the following parameters: denaturation at 94 °C for 30 s, annealing at 55 °C for 45 s and extension at 72 °C for 90 s, followed by a final extension at 72 °C for 10 min. The PCR product was diluted 1/1000 for use in LAMP (Table 1).

**Table 1:** Primers used for detection of phytoplasma DNA by LAMP designed from GenBank sequence KP053907 (1808 bp) *‘Cocos nucifera*’ phytoplasma clone 7–2 16S ribosomal RNA gene.

Primer	Sequence (5′-3′)	Position (bp)	Tm (°C)
Phyto1 F3	CGCCACATTAGTTAGTTGGTA	231	60.1
Phyto1 B3	TTCATCGAATAGCGTCAAGG	484	60.1
Phyto1 FIP	GTTTGGGCCGTGTCTCAGTGCCTACCAAGACGATGATG		
Phyto1 BIP	TACGGGAGGCAGCAGTAGGAGTACTTCATCGTTCACGC		
Phyto1LoopF	GTGGCTGTTCAACCTCTCA	308	62
Phyto1LoopB	AACTCTGACCGAGCAACG	376	62.1
BCS F3	GTAGCCTAACTACGCAAGTAG	1422	59.9
BCS B3	TCCTTCATCGGCTCTTAGT	1806	59.8
BCS FIP	CTTAGAAAGGAGGTGATCCATCCCGATCCGTCTAAGGTAGGGT		
BCS BIP	TTAGAGCACACGCCTGATAAGCCGAGTACCTTATGCTGGTG		
BCS LoopF	GGATACCTTGTTACGACTTAACC	1497	60.8
BCS LoopB	GTCGGTGGTTCAAGTCCAT	1653	62.1

The LAMP reactions were performed in a final volume of 25 µl consisting of 5 pM each of the F3 and B3 primers, 20 pM each of FIP and BIP primers, 10 pM each of LoopF1 and LoopB1 primers (5 µl in total), 15 µl of 1 × Isothermal Master Mix (OptiGene Ltd.) and 5 µl of template DNA. Both primer sets were optimized for amplification temperature and time on a subset of samples before processing the collection. After optimization, all reactions were performed using a Genie II (OptiGene Ltd.) for 45 min at 65 °C and 66.5 °C for primers Phyto1 and BCS primer sets respectively. This was followed by annealing at 98–80 °C (ramping at 0.05 °C-s) eactionn with no template DNA and/or a phytoplasma from a contrasting 16S group served as a negative control^21^. All samples were amplified in triplicate.

### Sequencing and phylogenetic analysis

Nested PCR positive products were sent to Biotech Corp (Biosune Co. Ltd., Shanghai, China) and Macrogen (Korea) for purification and sequencing. Phylogenetic analyses were performed for the coconut tissue and seedling trials separately using Geneious Prime 2023.2. Contigs for each sample were generated using Geneious assembler. Sequences for each experiment were aligned with the MAFFT Alignment (v7.490)^25^ using the ‘auto’ algorithm. Sequences were trimmed to a length of 1068 bp and the phylogenetic trees were constructed using the HKY genetic distance model and neighbor-joining method^26^ using 1000 replicates for bootstrap analysis.

## Results

### Detection of BCS phytoplasma in coconut tissues

Phytoplasma was detected from the extracted DNA from 10 husk samples (33.3%), five shell samples (16.7%), five flesh samples (16.7%), and four embryo samples (11.3%). Phytoplasma DNA was not detected from coconut milk or from the husk or flesh of betel nut (Table 2). The percentages of phytoplasma-positive tissues from the husk, shell, flesh and embryo of coconuts, did not differ significantly from one another (χ2 = 4.583, N = 120, df = 3, *P* = 0.205).

**Table 2:** Incidence of PCR detection of ‘*Ca*. P. noviguineense’ from tissues of coconut and betel nut drupes from field sites in Madang Province, PNG.

Tissue	Incidence of detection (proportion in brackets)			
	Mobdu	Siar	Bogia	Total
Coconut husk	4/10	3/10	3/10	10/30 (0.333)
Coconut shell	1/10	3/10	1/10	5/30 (0.167)
Coconut flesh	4/10	1/10	0/10	5/30 (0.167)
Coconut embryo	1/10	2/10	1/10	4/30 (0.113)

### Sequence and phylogenetic analysis for tissue samples (coconut)

**Figure 2:**
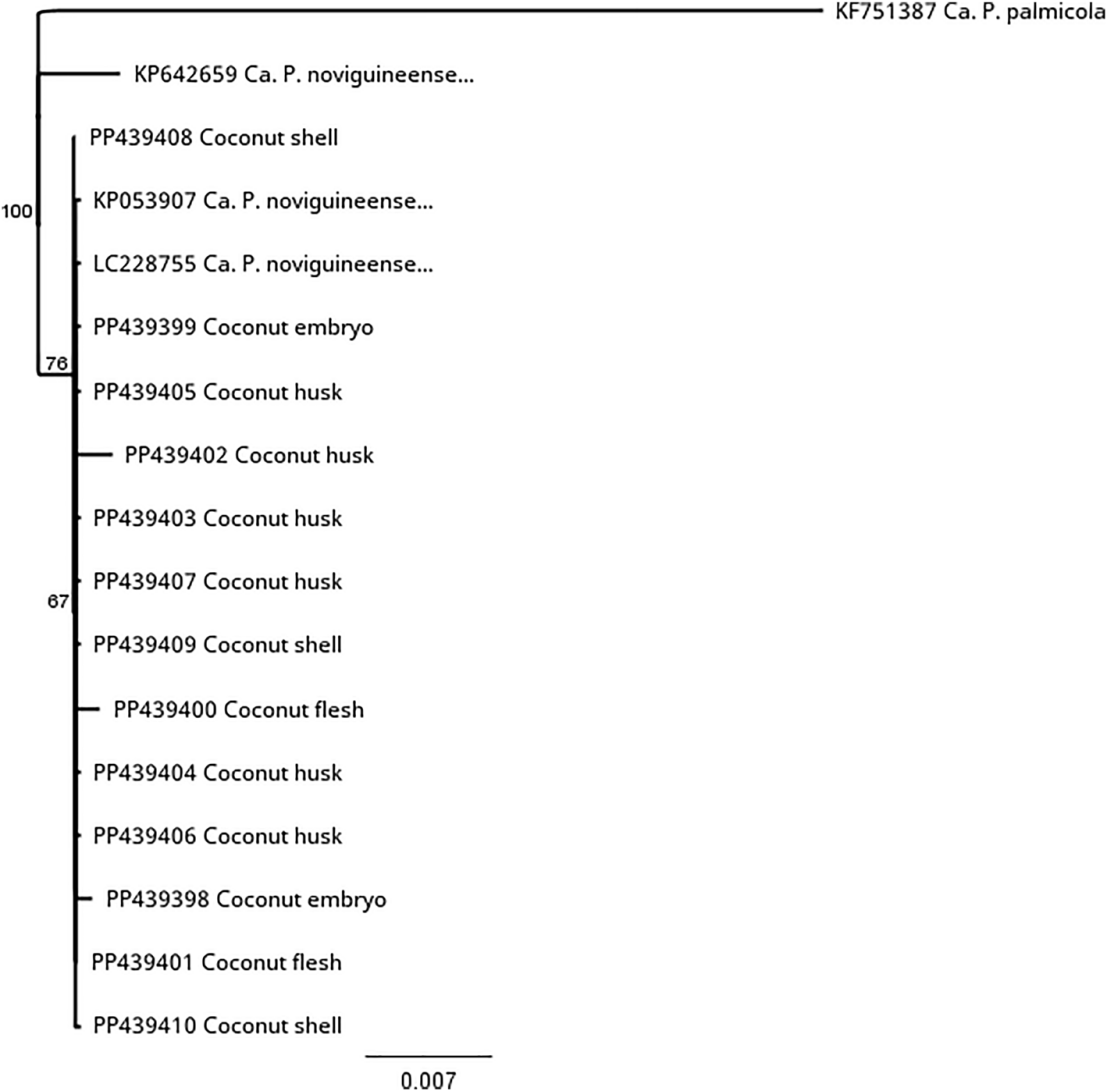
Consensus tree based on the partial 16S rRNA gene sequences of ‘*Ca*. P. noviguineense’ isolated from dissected coconut drupes using the Neighbor-joining method (Geneious Prime 2023.2.) Bootstrap values (> 50%) obtained from 1000 replicates are shown adjacent to the branches. ‘*Ca*. P. palmicola’ (KF51387) was used as the outgroup to root the tree, which also included KP642659 (BWAP, banana), KP053907 (BCS, coconut) and the reference strain LC228755 (‘*Ca.* P. noviguineense’, coconut). Bar, phylogenetic distance.

The phylogenetic tree based on the partial 16S rDNA sequences confirmed that the phytoplasma from the various coconut tissue to be members of 16SrIV phytoplasma group (Fig. 2). Sequence analysis showed that the phytoplasma DNA extracted from the coconut husk, shell, flesh and embryo had between 99.29 and 100% nucleotide identity with ‘*Ca*. P. noviguineense’ e.g. Bogia Coconut Syndrome phytoplasma and Banana Wilt Associated Phytoplasma strains (e.g. GenBank accession numbers LC228755, KP053907 and KJ460107).

‘*Ca*. P. noviguineense’ sequences from the various coconut tissues in this study were submitted to Genbank and have the following accession numbers: Coconut embryo (PP439398, PP439399), coconut flesh (PP439400, PP439401), coconut husk (PP439402-PP439407) and coconut shell (PP439408-PP439410). The coconut tissue samples typically had between 99.24 and 100% similarity to one another.

### Sequence and phylogenetic analysis for seed transmission samples (coconut and betel nut)

**Figure 3:**
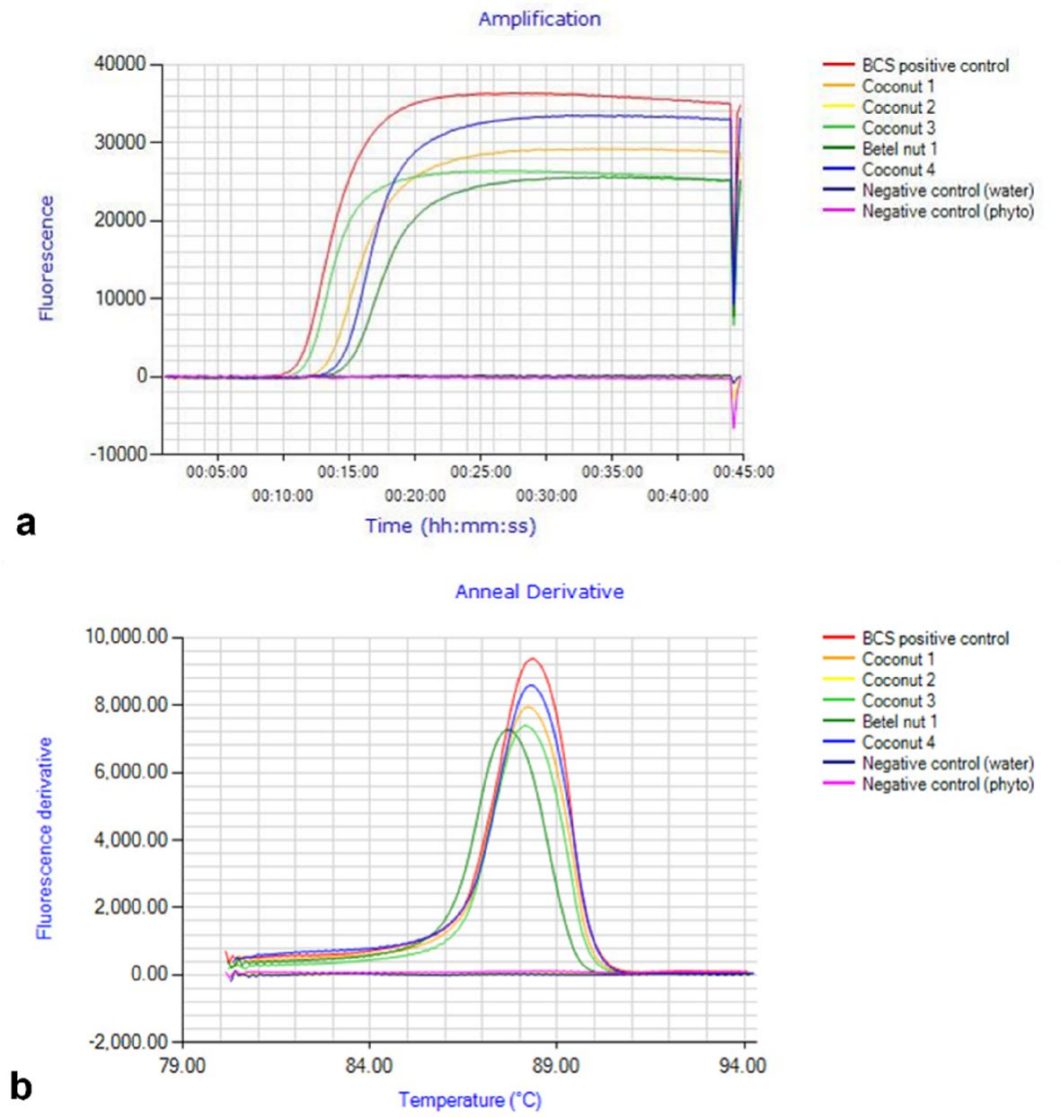
LAMP detection of BCS/BWAP in coconut and betel nut seedlings showing typical amplification and fluorescence of phytoplasma DNA using the BCS primer set (**a**) and typical melting curve observed (**b**).

Molecular testing of the leaf and petioles revealed seed transmission in coconut and betel nut. Both LAMP primer sets amplified BCS/BWAP phytoplasma in various samples, which was confirmed by nested PCR. When deploying the BCS LAMP primer set, the detection time (isothermal amplification shown as fluorescence) was rapid and occurred within 10–15 min (Fig. 3a). Typically, the melting curve for DNA amplified from coconut and betel nut tissue was between 88 and 88.5 °C (Fig. 3b).

**Figure 4:**
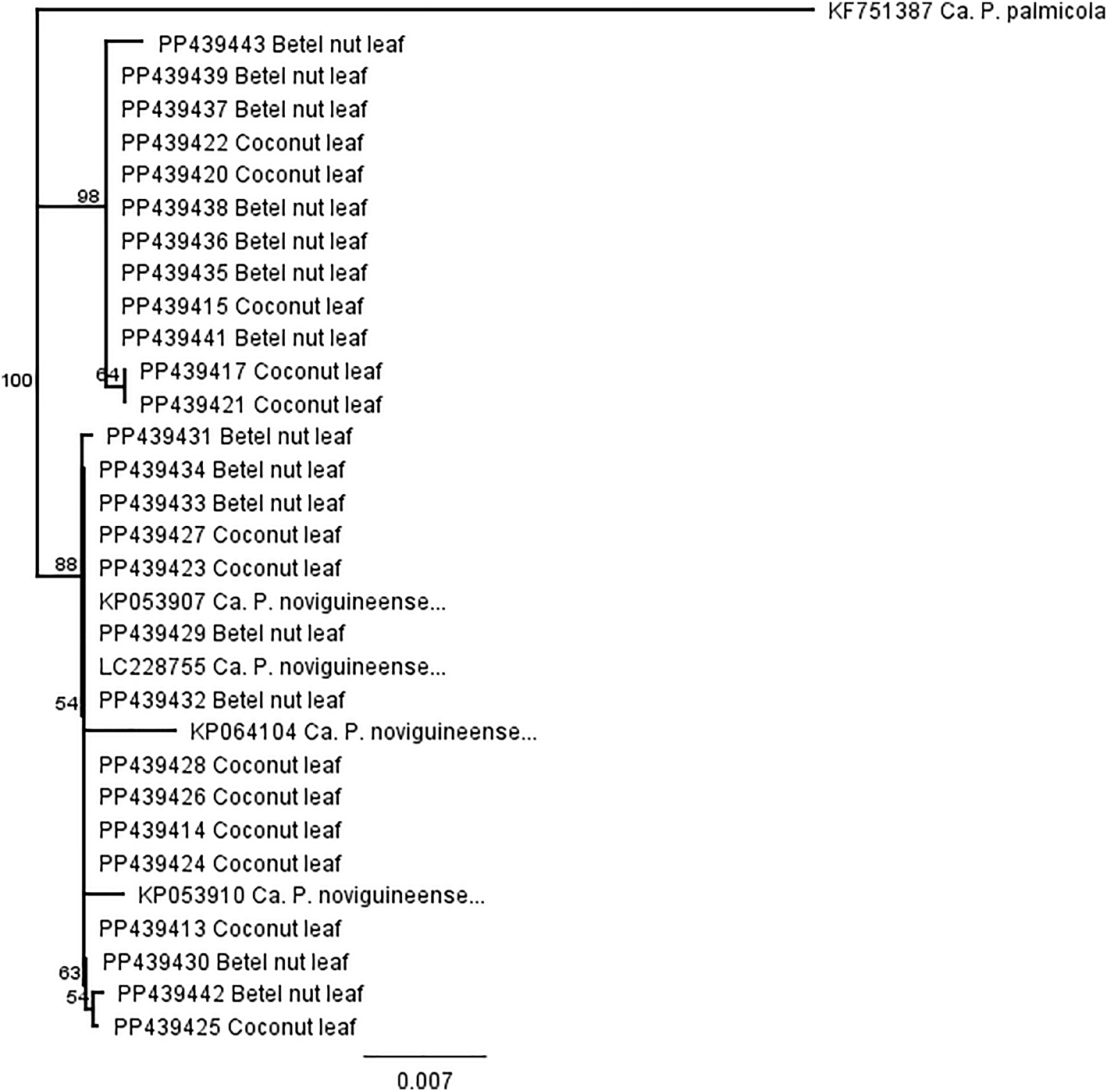
Consensus tree based on the partial 16S rRNA gene sequences of ‘*Ca.* P. noviguineense’ isolated from coconut and betel nut seedlings (leaf and petiole) using the Neighbor-joining method (Geneious Prime 2023.2.) Bootstrap values (> 50%) obtained from 1000 replicates are shown adjacent to the branches. ‘*Ca*. P. palmicola’ (KF51387) was used as the outgroup to root the tree, which also included KP053910 and KP064104 (BCS, betel nut), KP053907 (BCS, coconut) and the reference strain LC228755 (‘*Ca*. P. noviguineense’, coconut). Bar, phylogenetic distance.

Overall, 15 of 59 betel nut and 10 of 39 coconut seedling samples were found to be positive by nested PCR and LAMP. All samples showed very high sequence similarity to many ‘*Ca*. P. noviguineense’ sequences (97.8–99.6%) having GenBank accession numbers KP642659 (BWAP from banana in Debepari, PNG), LC228757 (BCS from coconut in Siar) or KP053901 (BCS from coconut in Madang) (Fig. 4).

‘*Ca*. P. noviguineense’ sequences from the coconut and betel nut seedlings in this study were submitted to Genbank and have the following accessions numbers: coconut leaf/petiole (PP439411-PP439428) including replicates of samples originating from the coconut genebank and betel nut (leaf/petiole) (PP439429-PP439443). The coconut samples shared between 99.17 and 100% similarity to one another and the betel nut samples shared between 99.09 and 100% similarity to one another.

‘*Ca.* P. noviguineense’ was detected from samples originating from Muad and Sugar villages and from 4 of the 18 samples collected from asymptomatic palms at the coconut genebank (thought to be outside the BCS-affected area) (Table 3). Caution is required in any future handling of planting material from this location especially when used as propagation material for other germplasm collections.

**Table 3:** Incidence of PCR detection of BCS DNA in coconut tissues collected in PNG.

No. samples	Genebank	Muad village	Oad village, Bilbil	Rivo village	Sugar village
Total	18	9	5	2	5
Positive	4	3	0	0	3

## Discussion

The results presented in this study provide evidence in support of seed transmission of BCS. Taken in conjunction with other studies of phytoplasma DNA in plant embryos and seedlings of various plant species^1,2^, these findings indicate that this mode of spread needs to be considered when formulating containment and management plans for BCS. Furthermore, there is a need for more detailed studies to better understand the relative importance of seed and insect vector transmission among the plant species that host BCS/BWAP.

Seed transmission of BCS/BWAP in the field is here demonstrated to be possible. There is now an urgent need for further study to determine the level of risk this poses to other areas of PNG that are still free of BCS. In other crops, seed production by phytoplasma infected plants is usually reduced^1,27^. In coconut lethal yellowing, one of the early symptoms is necrosis of the flowers and premature dropping of immature coconuts; however, timing of infection is known to be significant to seed viability. Observations during the research presented here revealed that mature coconuts were still found on infected palms^7,9^. In the case of BCS, although immature drupes begin to fall from infected palms during stage-1 symptoms, large drupes remain on trees that have progressed to stage-2 symptoms and these exhibit high levels of viability, producing shoots within three months of collection (S Woruba, personal observation). In contrast to annual crops, Nipah et al.^12^ found increased germination rates in seeds from diseased palms compared to healthy palms. Oropeza et al.^18^ reported a detection rate of 11% (nested PCR) and 32% (qPCR) for embryos and 42% of plantlets grown in vitro, although after 18 months when the surviving plantlets were tested, the positive detection rate dropped to 39% (qPCR) and 29% (nested PCR). The detection rate in seedlings in this work was lower with 25% (10/39) coconut seedlings positive for ‘*Ca*. P. noviguineense’. In other studies on seed transmission, the detection rate of phytoplasma in seedlings decreases with time^1^. The LAMP method described here provided a rapid detection of ‘*Ca. *P. noviguineense’ in coconut and betel nut tissues, significantly reducing the time to screen samples. To satisfy quarantine regulations in Australia, DNA was extracted from samples in PNG prior to LAMP processing in Australia. Future work could optimize extraction protocols from plant tissue in PNG to enable rapid and inexpensive methods like LAMP to be employed in PNG.

Coconut can take up to seven years to produce fruits so it is questionable whether ‘*Ca*. P. noviguineense’ infected seedlings would survive long enough to be problematic (though all of the seedings grown in this study survived at 12 months of age). A higher risk is that ‘*Ca.* P. noviguineense’ would be acquired from these young plants by any of the putative vectors that have been identified when feeding on infected seedlings^21^. The epidemiology of the phytoplasma-vector-plant-habitat is still under investigation. In Florida, where the American palm cixiid (*Haplaxius crudus*) is the vector of coconut lethal yellowing, adults prefer mature palms and rarely feed on immature palms^28,29^. In PNG, BCS symptoms have been observed in palms of all ages and further studies need to be conducted on vector species to determine if they feed on coconut and betel nut seedlings. There is evidence in other phytoplasma pathosystems that vectors are highly attracted to infected plants^30^.

Although the rate of phytoplasma seed transmission is low, there remains a significant risk of movement to new areas currently free of the disease^1^. This is especially relevant in PNG where BCS is currently restricted to the Madang province (BWAP has been recorded in the Markham Valley, Morobe province). To date, best practice from managing coconut lethal yellowing remains sanitation and replacement with resistant varieties, which so far, are yet to been identified in PNG^8,31,32^.

## Conclusions

Further studies are urgently required to confirm the finding of this study on seed transmission of ‘*Ca. *P. noviguineense’, which has important implications for food security in PNG. Current phytosanitary arrangements prohibit the movement from Madang Province of all vegetative planting material of all palm species and require that coconuts are de-husked. The present findings suggest that de-husking, though it removes green tissues that could be fed upon by a vector, does not remove all tissues to contain phytoplasma DNA. Complete prohibition of movement of coconuts would be necessary to avoid the risk of seed transmission and disease spread. Further, the large volume of trade in fresh betel nut including from Madang Province (where it grows readily) to the Highlands (where it is highly valued as a mild narcotic) means that this work is urgent. Though use of betel nut involves its destruction (by chewing) rather than it being sown, there is a risk that lost or discarded drupes produce shoots that could be infected and are fed upon by vectors.

## Data Availability

Sequence data that support the findings of this study have been deposited in Genbank with the accession codes used in Figs. 2 and 4.
